# Covid-19 and the pandemic of fear: reflections on mental health

**DOI:** 10.11606/s1518-8787.2020054002486

**Published:** 2020-04-28

**Authors:** Dandara Almeida Reis da Silva, Rodrigo Fernandes Weyll Pimentel, Magno Conceição das Merces

**Affiliations:** I Universidade do Estado da Bahia Departamento de Ciências da Vida SalvadorBA Brasil Universidade do Estado da Bahia. Departamento de Ciências da Vida. Salvador, BA, Brasil; II Universidade Federal da Bahia Complexo Hospitalar Professor Edgard Santos SalvadorBA Brasil Universidade Federal da Bahia. Complexo Hospitalar Professor Edgard Santos. Salvador, BA, Brasil

Covid-19 is a respiratory infection caused by coronavirus- initially detected in China, in December 2019, which progresses to pneumonia in 81% of the cases, with an average fatality rate of 2.3%^1^. It was declared a pandemic by the World Health Organization on March 11, 2020, almost ninety days after the first cases appeared. With high transmissibility, vertiginous increase in the number of cases, and clinical severity, it is impossible to disregard its psychological effects.

Despite being a recent pathology, studies addressing the theme of emotional stress in today’s scenario already emerged. The bibliographic research conducted on March 22, 2020, using the search strategies “Psychological Stress AND covid 19”, “Estresse Psicológico AND covid 19”, and “mental disorder AND covid 19” in Pubmed and BIREME, found seven articles, all with equivalence in both databases. No other filters have been defined. Two articles in Chinese were not evaluated. After reading the other five articles in full, two were originals; the others represented brief communications.

Both original articles with primary data were conducted in China. One of these papers evaluated three groups: general population, frontline nurses and general nurses ^2^. Frontline nurses were the least affected by emotional stress, condition linked to strategies to cope with emotional stress developed by these professionals. However, the accumulation of work overload may change this scenario, and the continuous exposure to emotional stress may trigger other disorders^3^. Regardless of the group, emotional stress was present in more than 60%^2^. In the general population (1210 individuals), 28.8% exhibited symptoms of moderate to severe anxiety and 53.8% reported psychological impact of moderate to severe intensity, because of the epidemic^4^.

Considering the epidemiological behavior of covid-19, the period of social isolation may be necessary indefinitely. Regardless of the covid-19 pandemic, social isolation itself is capable of triggering psychotic symptoms, as Kellerman et al. described in 1977^5^. In this study, they evaluated individuals in social isolation due to oncological disease, and observed symptoms of depression (92.3%), anxiety (76.9%), sleep disorders (61.5%) and hallucinations in 38.5% of the subjects, among other alterations^5^.

Besides the covid-19 pandemic, health professionals and the general population are at risk of mental illness. We must develop psychological support strategies to promote mental health, emphasizing the use of online technologies to maintain the necessary physical distancing. Keep in mind those already suffering from psychiatric disorders, which can be worsened by the current scenario. Surveillance will be needed to control the “pandemic of fear” and treat mental illness even after the covid-19 pandemic is resolved.
